# Idiopathic Myointimal Hyperplasia of the Mesenteric Veins: Diagnosed After Resection due to Insufficient Palliative Surgery

**DOI:** 10.70352/scrj.cr.25-0129

**Published:** 2025-06-07

**Authors:** Natsuki Hoshino, Jun Yamamoto, Nao Obara, Shogo Takei, Eiichi Nakao, Yasuhiro Shimizu, Yusaku Tanaka, Taichi Yabuno, Hiroyuki Hayashi, Yasuhisa Mochizuki

**Affiliations:** 1 Department of Gastrointestinal Surgery, Yokohama Municipal Citizen’s Hospital, Yokohama, Kanagawa, Japan; 2 Department of Inflammatory Bowel Disease, Yokohama Municipal Citizen’s Hospital, Yokohama, Kanagawa, Japan; 3 Department of Pathology, Yokohama Municipal Citizens Hospital, Yokohama, Kanagawa, Japan

**Keywords:** idiopathic myointimal hyperplasia of mesenteric veins, ischemic colitis, colostomy, colectomy

## Abstract

**INTRODUCTION:**

Idiopathic myointimal hyperplasia of the mesenteric veins (IMHMV) is a rare, non-thrombotic ischemic bowel disease caused by the proliferation of smooth muscle cells in the venous intima. Most patients are initially diagnosed with typical ischemic colitis or inflammatory bowel disease (IBD) and are treated nonsurgically. There is no established treatment for IMHMV, and surgery is the mainstay of treatment, although the optimal surgical approach remains unclear.

**CASE PRESENTATION:**

A 66-year-old man visited the previous hospital with abdominal pain and was clinically diagnosed with ischemic colitis. Diagnostic colonoscopy showed inflammation from the sigmoid colon to the lower rectum. The biopsy did not reveal malignancy or IBD. His clinical condition did not improve after 2 months of conservative treatment, and he underwent a diverting colostomy. The symptoms temporarily improved, and he was referred to our hospital. However, 1 month postoperatively, computed tomography showed a rupture of the sigmoid colon and fluid collection around the sigmoid colon. He was diagnosed with perforation of the sigmoid colon due to relapse of ischemic colitis and underwent Hartmann’s procedure. Histopathological examination showed stenosis and obstruction of the venous lumen at the perforation site with whole-layer necrosis, and proliferation of smooth muscle cells in the venous intima. The pathological diagnosis was IMHMV. He was discharged from our hospital 23 days after surgery without major complications and has had no symptoms or recurrence 8 months after surgical resection.

**CONCLUSIONS:**

IMHMV is a rare disease, infrequently suspected preoperatively and typically diagnosed after surgical resection. Palliative surgery, such as colostomy, may not be a sufficient treatment for IMHMV.

## Abbreviations

IBD
: inflammatory bowel disease
IMHMV
: idiopathic myointimal hyperplasia of mesenteric veins
MIVOD
: mesenteric inflammatory occlusion of mesenteric veins
SMA
: smooth muscle antibody
SLE
: systemic lupus erythematosus

## INTRODUCTION

IMHMV is an extremely rare disease in which smooth muscle proliferation in the intima of mesenteric veins causes zonal intestinal ischemia.^1)^ The clinical features of IMHMV are similar to those of IBD. Although the clinical course of IMHMV is relatively long due to its refractoriness to medical therapy, it is difficult to diagnose IMHMV without histopathological examination of a resected specimen.^2)^ We herein report the case of a patient with IMHMV who eventually required surgical resection due to inadequate response to colostomy.

## CASE PRESENTATION

**Fig. 1:**
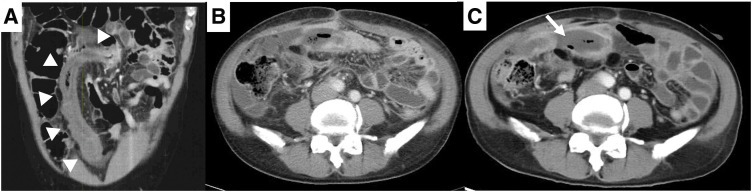
(**A**) CT at the initial examination at the previous hospital. The patient had wall thickening from the rectum to the sigmoid colon (white arrowhead). (**B**) CT at the last examination after colostomy at the previous hospital. Enteritis had worsened, and ascites had increased. (**C**) CT before colectomy at our hospital. There was edematous wall thickening from the sigmoid colon to the rectum, with partial wall disruption in the sigmoid colon. Peritoneal fluid accumulation with encapsulation (white arrow), peritoneal thickening, and increased density of abdominal adipose tissue were observed.

**Fig. 2:**
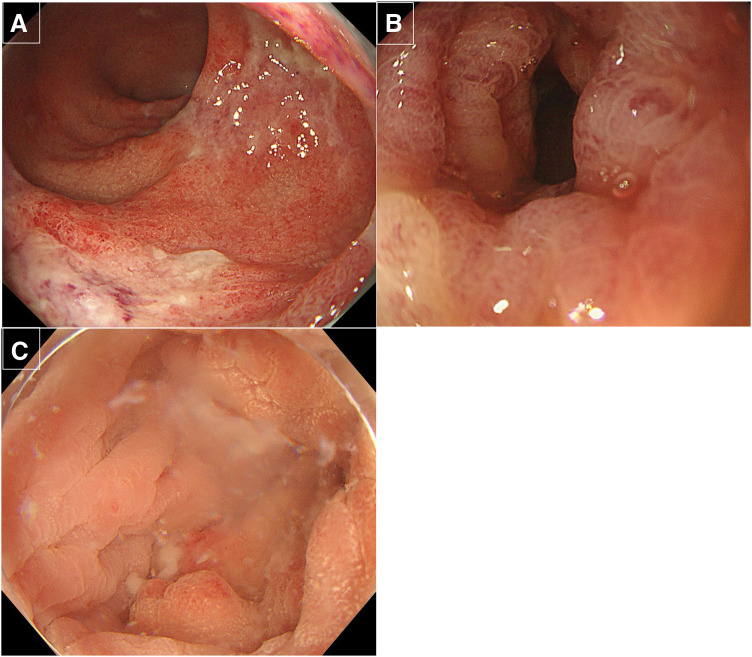
Colonoscopy at the previous hospital (**A**, **B**) and our hospital (**C**). (**A**) Coarse mucosa suspected of ischemia was observed from the rectum to 20 cm from the anal verge. (**B**) Edema of the intestinal mucosa was continuous from 20 to 35 cm from the anal verge. Scope passage was possible, and no significant ulcerative changes were observed. (**C**) The mucosa was relatively soft with scattered inflammatory polyps; however, active inflammation was negative up to 10 cm from the anal verge.

A 66-year-old male, who is medicated with bisoprolol fumarate and edoxaban for ventricular extrasystole and internal carotid thrombosis, presented with abdominal pain and was diagnosed with ischemic colitis at a previous hospital. Although he received dietary restrictions and antibiotics for 2 months, his symptoms did not improve and he was hospitalized at a previous hospital. Contrast-enhanced CT demonstrated wall thickening and poor enhancement of the sigmoid colon and rectum (**Fig. 1A**). Occlusion or stenosis of the inferior mesenteric artery was not observed. Diagnostic colonoscopy showed extensive inflammation from the sigmoid colon to the lower rectum (**Fig. 2A**–**2C**). Biopsies did not show the findings of malignancy or IBD. The inflammation spread extensively from the descending colon to the lower rectum despite the dietary restrictions and antibiotics; therefore, he underwent a transverse colostomy. The clinical condition temporarily improved and he was discharged from the previous hospital. However, he developed a fever and serum inflammatory markers were elevated 1 month after the colostomy. CT demonstrated relapse of colitis and increased ascites (**Fig. 1B**). He was referred to our department due to his relocation.

**Fig. 3:**
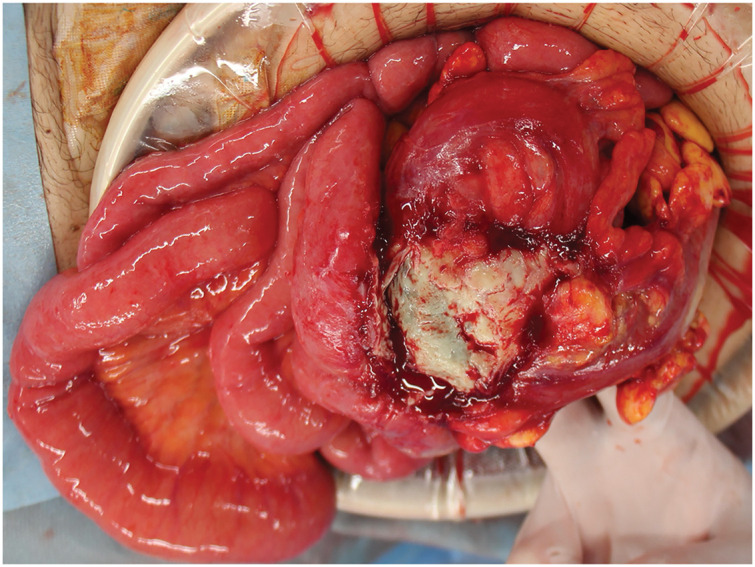
Intraoperative findings. The wall of the sigmoid colon was partially necrotic, perforated, and adhered to the small intestine.

Laboratory data showed high levels of inflammatory markers despite medical treatment. The contrast-enhanced CT scan (**Fig. 1C**) performed at our hospital showed edema from the sigmoid colon to the rectum and wall disruption in the sigmoid colon, surrounded by encapsulated fluid collection with the dirty fat sign. He was diagnosed with perforation of the sigmoid colon with intra-abdominal abscess and underwent Hartmann’s procedure (**Fig. 3**) the following week.

**Fig. 4:**
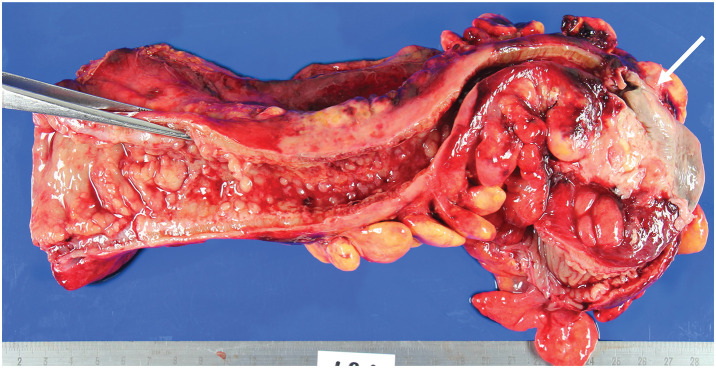
Macroscopic findings. A necrotic area of mucosa (white arrow) was located 9 cm anorectally from the oral margin, and perforation into the mesentery was observed. On the anorectal side, there was a 1 cm thickened wall and multiple inflammatory polyps over a length of about 15 cm, and the lumen was narrowed to about 3 cm.

**Fig. 5:**
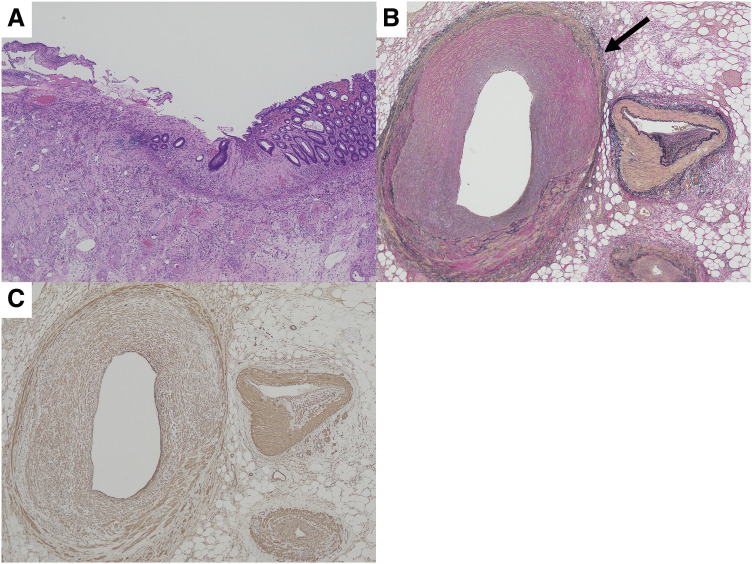
Histopathological findings. (**A**) Hematoxylin–eosin staining (×40). Full-thickness necrosis at the perforation site and a mucosal defect around the perforation site were seen. There was no inflammatory cell response. (**B**) Elastica van Gieson staining (×100). Intimal thickening of the vein and a narrow vessel lumen (black arrow) were observed. (**C**) Immunohistochemical staining for SMA (×100). The intimal thickening contained numerous SMA-positive cells and smooth muscle had invaded and proliferated into the intima (myointimal hyperplasia). SMA, smooth muscle antibody

Macroscopically, the necrotic area of the mucosa, which was located 9 cm from the oral margin, was perforated into the mesentery (**Fig. 4**). On the anorectal side, there was a 1 cm thickened wall and multiple inflammatory polyps over a length of about 15 cm, and the lumen was narrowed to about 3 cm. Histopathological examination showed full-thickness necrosis at the perforation site and a mucosal defect around the perforation site. The inflammatory cell response was poor, and the necrosis was not likely due to infection but rather due to ischemia. The lumen of the vessel was extremely narrow at the perforation site, and Elastica van Gieson staining confirmed the intimal thickening of the vein. The intimal thickening contained numerous SMA-positive cells, and smooth muscle had invaded and proliferated into the intima (myointimal hyperplasia) (**Fig. 5**). These findings were consistent with IMHMV. The patient was discharged 23 days after colectomy without major complications. One year after the operation, he was well without recurrence.

## DISCUSSION

Ischemic colitis is an ischemic bowel disease caused by transient ischemia of the arterial blood supply to the colon. Unlike superior mesenteric artery thrombosis, ischemic colitis does not cause organic vascular occlusion. In 1963, Boley et al.^3)^ proposed the concept of “reversible vascular occlusion of the colon,” a disease in which ischemia occurs due to reversible vascular occlusion of the colon. In 1966, Marston et al.^4)^ called the disease “ischemic colitis” and classified it into gangrene, stricture, and transient types (Marston classification). Later, Marston revised the definition of ischemic colitis to exclude the gangrene type, but the classification including is still widely used.^5)^

Most intestinal ischemic diseases, such as ischemic colitis, are due to arterial ischemia, while rare cases of venous ischemia are often thrombotic.^6)^ Rare non-thrombotic mesenteric venous occlusion resulting in bowel ischemia due to venous sclerosis or phlebitis has also been reported.^7)^ Non-thrombotic mesenteric vein occlusion in vasculitis such as SLE and Behçet’s disease, as well as intestinal lymphocytic phlebitis, have been reported and are collectively referred to as MIVOD.^8)^

IMHMV is considered a non-thrombotic, non-inflammatory ischemic bowel disease with irreversible venous obstruction caused by smooth muscle proliferation in the venous intima, and was first described by Genta and Haggitt in 1991.^1)^ It is more common in healthy young men and tends to occur in the sigmoid colon of the rectum.^9)^ Clinical symptoms include bloody stools, abdominal pain, and weight loss, and the diagnosis is difficult because the disease can easily be misdiagnosed as an inflammatory disease such as IBD, which has the same clinical features.^10)^ In the absence of clear histologic diagnostic criteria, a biopsy can rarely distinguish ischemic changes from known IBD, and a definitive diagnosis is possible only after surgery.^2)^ However, Abu-Alfa et al.^11)^ described mucosal findings in biopsies and resection specimens from a patient with IMHMV, noting the presence of thick-walled hyalinized vessels, and these vascular alterations were absent in other types of ischemic enterocolitis and mucosal prolapse. There have been reports indicating that preoperative diagnosis can be made based on mucosal biopsy findings. These reports have focused on vascular alternations such as fibrin deposits and myointimal thickening of small mucosal vessels, as well as superficial mucosal necrosis and lamina propria fibrosis with dilated, thick-walled capillaries containing fibrin thrombi.^12,13)^ Furthermore, a recent study reported that “arteriolized” capillaries, subendothelial fibrin deposits, and perivascular hyalinization are common and specific features that may aid in the identification of IMHMV in biopsy samples.^14)^ Pathologists should consider IMHMV when these findings are present. In this case, the endoscopic biopsy revealed typical characteristics of ischemic colitis, such as hyalinized stroma, bleeding mucosa, and minimal inflammatory cell infiltration, and there were no changes noted as mentioned earlier. As a result, diagnosing IMHMV preoperatively was challenging.

Pathologically, IMHMV is characterized by the proliferation of intimal smooth muscle with the thickening of mostly small- and medium-sized mesenteric veins, resulting in luminal obstruction and ischemic changes of the mucosa.^2)^ Compared with other inflammatory diseases, IMHMV is not caused by arterial thromboembolism, venous thrombosis, or vasculitis, and its etiology remains poorly understood.^15)^ In the present case, there was no evidence of phlebitis in the histopathologic findings, making it easy to distinguish IMHMV from MIVOD. Lincango et al.^16)^ summarized a review of 88 patients with IMHMV. They reported that 82% of the patients were male and relatively young, with a mean age of 56.6 years. Rectal and sigmoid colon involvement accounted for 81% of cases. Only 1 patient achieved remission with medical therapy, and 99% of the other patients required surgery. The most common procedures were Hartmann’s procedure (24%) and partial colectomy (19%). Ninety-nine percent of patients achieved remission with resection of the lesions. This is the first reported case in which a colostomy was performed prior to resection of the lesion. Although colostomy is usually considered a treatment for ischemic enteritis that does not improve with conservative management, in IMHMV, which has a different pathophysiology, an in-office procedure such as colostomy may not be sufficient. However, pathologic diagnosis of IMHMV without lesion resection is extremely rare. Therefore, it is important not to hesitate to consider the possibility of IMHMV and to perform bowel resection in cases of ischemic enteritis that do not improve with colostomy. In patients with suspected ischemic bowel disease, it is important to determine the appropriate timing of surgical treatment as well as conservative therapy, and to determine the appropriate surgical technique to improve the patient’s prognosis.

Kikuchi et al.^17)^ identified the surgical indications for ischemic colitis as necrotic perforation, massive hematochezia, and peritonitis during the early stage of disease onset, as well as prolonged inflammation and stenosis occurring later, with the 5th week post-onset being a suitable time for intervention. According to this timeline, the first 5 weeks following symptom onset are crucial for assessing the need for surgery or identifying other potential conditions in suspected cases of ischemic bowel disease. It is essential to consistently monitor both subjective symptoms and inflammation markers. On the other hand, Li et al. summarized 70 cases of IMHMV and reported that the average duration between symptom onset and surgery was 4.5 months.^18)^ The primary indications for surgery included abdominal pain, hematochezia, or persistent symptoms. More than half of the patients (37/70) were incorrectly diagnosed with IBD and given medication, which may have postponed their surgery. They also reported that complications such as perforation or hematochezia were mainly caused by IBD-related medication, with perforation and bleeding often occurring after steroid use. Reducing misdiagnosis of IBD in IMHMV patients may be an issue in the future. It is critical to minimize unnecessary treatment and expedite surgery, thereby reducing complications.

## CONCLUSIONS

We described a rare case of IMHMV that was diagnosed after resection because palliative surgery was insufficient.

## DECLARATIONS

### Funding

This study received no funding.

### Authors’ contributions

All authors contributed to the diagnosis and treatment of the patient.

NH drafted the manuscript.

JY revised the manuscript.

All authors read and approved the final manuscript.

### Availability of data and materials

Not applicable.

### Ethics approval and consent to participate

This work does not require ethical considerations or approval. Informed consent to participate in this study was obtained from the patient.

### Consent for publication

Patient consent was obtained for the publication of this case report.

### Competing interests

The authors declare that they have no competing interests.
